# Multistrand Twisted Triboelectric Kevlar Yarns for Harvesting High Impact Energy, Body Injury Location and Levels Evaluation

**DOI:** 10.1002/advs.202401076

**Published:** 2024-03-15

**Authors:** Fangjing Xing, Xiaobo Gao, Jing Wen, Hao Li, Hui Liu, Zhong Lin Wang, Baodong Chen

**Affiliations:** ^1^ Beijing Institute of Nanoenergy and Nanosystems Chinese Academy of Sciences Beijing 101400 P. R. China; ^2^ School of Nanoscience and Engineering University of Chinese Academy of Sciences Beijing 100049 P. R. China; ^3^ School of Materials Science and Engineering Inner Mongolia University of Technology Hohhot 010051 P. R. China; ^4^ Changchun University of Chinese Medicine Jilin 130117 P. R. China; ^5^ Georgia Institute of Technology Atlanta GA 30332 USA

**Keywords:** harvesting high impact energy, multistrand twisted triboelectric Kevlar, self‐powered system, triboelectric nanogenerators

## Abstract

Developing ultrahigh‐strength fabric‐based triboelectric nanogenerators for harvesting high‐impact energy and sensing biomechanical signals is still a great challenge. Here, the constraints are addressed by design of a multistrand twisted triboelectric Kevlar (MTTK) yarn using conductive and non‐conductive Kevlar fibers. Manufactured using a multistrand twisting process, the MTTK yarn offers superior tensile strength (372 MPa), compared to current triboelectric yarns. In addition, a self‐powered impact sensing fabric patch (SP‐ISFP) comprising signal acquisition, processing, communication circuit, and MTTK yarns is integrated. The SP‐ISFP features withstanding impact (4 GPa) and a sensitivity and response time under the high impact condition (59.68 V GPa^−1^; 0.4 s). Furthermore, a multi‐channel smart bulletproof vest is developed by the array of 36 SP‐ISFPs, enabling the reconstruction of impact mapping and assessment of body injury location and levels by real‐time data acquisition. Their potential to reduce body injuries, professional security, and construct a multi‐point personal vital signs dynamic monitoring platform holds great promise.

## Graphical abstract

A self‐powered impact sensing fabric patch (SP‐ISFP) is proposed that works effectively even at impacts of up to 4 GPa. By placing a series of SP‐ISFPs in a bulletproof vest, it can reconstruct a human impact map and assess the extent of damage through data acquisition, providing a reliable safety net for individuals in high‐risk environments.

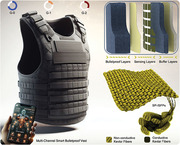

## 1 Introduction

Personal safety and security are of great importance to human society.^[^ ^1^ ^]^ Protective clothing, as an important part of personal protective wearables, plays a vital role in the electronic industry,^[^ ^2^ ^]^ medical service,^[^ ^3^ ^]^ firefighting,^[^ ^4^ ^]^ and private security.^[^ ^5^ ^]^ Ideal protective clothing should not only be able to protect the human body from injury but also have intelligent functions such as monitoring physiological signals and detecting potential hazards.^[^ ^6^ ^]^ However, the mechanical properties of current traditional rescue electronics are weak and the devices are prone to destroy under impact. In this case, self‐powered, impact‐resistant electronic equipment is urgently needed.

In recent times, there has been a growing interest in integrating electronics with textiles.^[^ ^7^ ^]^ Fabric‐based triboelectric nanogenerators (TENGs) offer a convenient means to convert mechanical energy from human movement into electricity.^[^ ^8^ ^]^ These fabric‐based TENGs possess several advantageous qualities, including lightweight,^[^ ^9^ ^]^ flexibility,^[^ ^10^ ^]^ breathability,^[^ ^11^ ^]^ washability,^[^ ^12^ ^]^ and the ability to detect microscale motion,^[^ ^13^ ^]^ and capture subtle physiological signals from the human body,^[^ ^14^ ^]^ as a result, they have emerged as a viable power source for wearable electronic devices,^[^ ^15^ ^]^ garnering global attention. Currently, there is ongoing research focusing on smart wearable devices that not only enable real‐time monitoring of users’ vital signs,^[^ ^16^ ^]^ but also incorporate emergency alarm systems to provide timely assistance and rescue when needed.^[^ ^17^ ^]^ These advancements aim to enhance personal safety and well‐being, ensuring prompt response in critical situations. Despite the advancements in smart wearable devices, there are still challenges related to the weak tensile and shear resistance of the materials used and the insufficient mechanical properties of the sensing systems. These limitations make the devices susceptible to damage upon impact, compromising their ability to adequately protect the human body in high‐impact scenarios.

Compared with ordinary fabric, high‐performance polymer textiles, represented by Kevlar, are an indispensable component of protective clothing due to their excellent mechanical performance.^[^ ^18^ ^]^ The bulletproof vest, which is made of high‐strength synthetic aramid fiber (Kevlar fiber),^[^ ^19^ ^]^ because of its bulletproof effect,^[^ ^20^ ^]^ light weight^[^ ^21^ ^]^ and comfortable wear,^[^ ^22^ ^]^ is popular in many countries.^[^ ^23^ ^]^ Over the past decade, considerable research efforts have been devoted to the development of Kevlar‐based triboelectric nanogenerators. Notable examples include Nanofibrous Kevlar Aerogel,^[^ ^21^ ^]^ Janus Graphene/Kevlar,^[^ ^22^ ^]^ functional STG/Kevlar composites,^[^ ^24^ ^]^ and Kevlar/Epoxy based TENG.^[^ ^25^ ^]^ These innovative approaches aim to harness the unique properties of Kevlar to enhance the performance and functionality of triboelectric nanogenerators. Indeed, the existing methods for developing Kevlar‐based smart bulletproof vests often involve complex and time‐consuming preparation processes, which can negatively impact the material's tensile strength and flexibility. Therefore, it remains challenging to create a Kevlar‐based smart bulletproof vest using simple and straightforward techniques.

In this study, we used conductive and non‐conductive Kevlar fibers to produce multistrand twisted triboelectric Kevlar (MTTK) yarn through a simple twisting process, which can ensure flexibility and does not affect the tensile strength (372 mPa), in which conductive Kevlar fibers serving as the conductors for electronic transmission and non‐conductive Kevlar fibers acting as the friction shell, this unique design ensures the durability and strength of the MTTK yarn. Self‐powered impact sensing fabric patch (SP‐ISFP) woven from MTTK yarn can withstand impact of up to 4 GPa, while the SP‐ISFPs array is capable of converting various impact forces (such as daggers, fists, and clubs) into electrical signals without requiring an external power source. Building upon this, we have developed a multi‐channel smart bulletproof vest that encodes the pulse signals into color mapping to indicate the location and pressure level of the impact, which can be displayed on a terminal. In summary, this research measures and visualizes impactors impacts, offering a reliable method for rescue assistance and facilitating in‐depth analysis.

## 2 Results and Discussion

**Figure 1:**
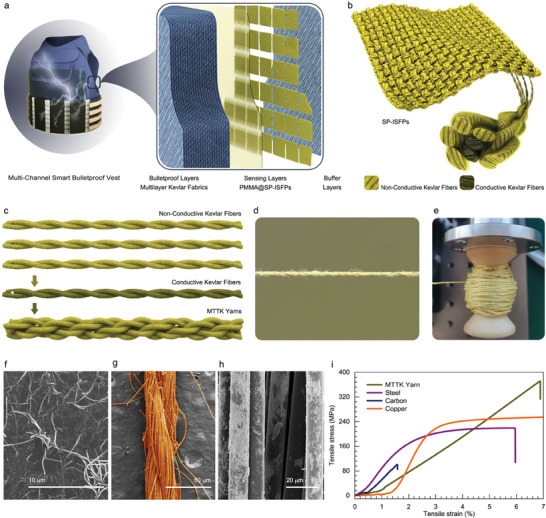
Structural design of multi‐channel smart bulletproof vest and MTTK yarn. a) Schematic diagram and internal structure of smart bulletproof vest, including bulletproof layer, sensing layer, and buffer layer. b) The schematic drawing of SP‐ISFP. c) The fabrication process and structural design of MTTK yarn. d,e) Optical photograph of MTTK yarn. f) SEM image of silver nanowires. g,h) Surface morphology SEM image of conductive Kevlar fibers. i) Stress‐strain curves of MTTK yarn, Steel yarn, Carbon yarn, and Copper yarn with the same diameter.

The morphological characteristics of impact injury vary with impact velocity, surface morphology of impactor and impact site of the human body, including epidermal exfoliation, subcutaneous bleeding, soft tissue laceration, visceral rupture, and fracture. A multi‐channel smart bulletproof vest is designed, shown in **Figure** **1**a. The multi‐channel smart bulletproof vest consists of a clothing cover, a bulletproof layer, a buffer layer, and a sensor layer. In particular, the bulletproof layer is typically made up of multiple layers of high‐strength and high‐modulus fiber fabrics (Kevlar), this layer serves to deflect or absorb the impact of incoming bullets or explosive fragments. The buffer layer (closed‐cell knitted composite cloth, acrylic sheet, and soft polyurethane foam) is employed to dissipate the kinetic energy generated from impact and minimize non‐penetrating damage. The sensor layer consists of the array of 36 SP‐ISFPs (Figure 1b), can convert the impact force of different impactors (such as daggers, fists, clubs) into electrical signals without the need for a power supply, so as to detect and evaluate the impact position and impact grade of the body, thereby providing a dependable means for rescue assistance and facilitating further analysis. The SP‐ISFP is woven from MTTK yarns by plain weave, Figure 1c and Figure S1 (Supporting Information) show a schematic of the fabrication process of MTTK yarn, where conductive Kevlar fibers as the electronic transmission core is tightly wound by the non‐conductive Kevlar fibers interweaving with each other. By utilizing the multiaxial high‐speed cord braiding machine, the production of hundreds of meters of MTTK yarn becomes feasible and reliable (refer to Movie S1, Supporting Information). Optical images of the MTTK yarn are depicted in Figure 1d‐e, demonstrating its excellent flexibility akin to ordinary fibers. It can be bent, knotted, and has a diameter of ≈1 mm (Figure S2, Supporting Information). The scanning electron microscope (SEM) image of conductive Kevlar fibers is shown in Figure 1f–h. It can be seen from Figure 1h that silver nanowires adhere to each single yarn of the conductive Kevlar fibers, and the slight aggregation and dispersion on a single yarn will not affect the electrical conductivity of the whole Kevlar fiber. At the same time, the electrical conductivity and resistivity of the conductive Kevlar fibers were also tested, which were 6.95 × 10^−3^ s m^−1^ and 1.44 × 10^2^ Ω m, respectively. To characterize the mechanical properties of different materials, including MTTK yarn, steel, carbon fiber, and copper (same diameter:1 mm), their tensile stress‐strain curves were measured using a universal testing machine. The results are presented in Figure 1i. From the figure, it can be observed that compared to the other materials, MTTK yarn exhibits the highest mechanical strength. Its corresponding tensile strength measures at 372 MPa, which is nearly three times that of commercial carbon fibers.

**Figure 2:**
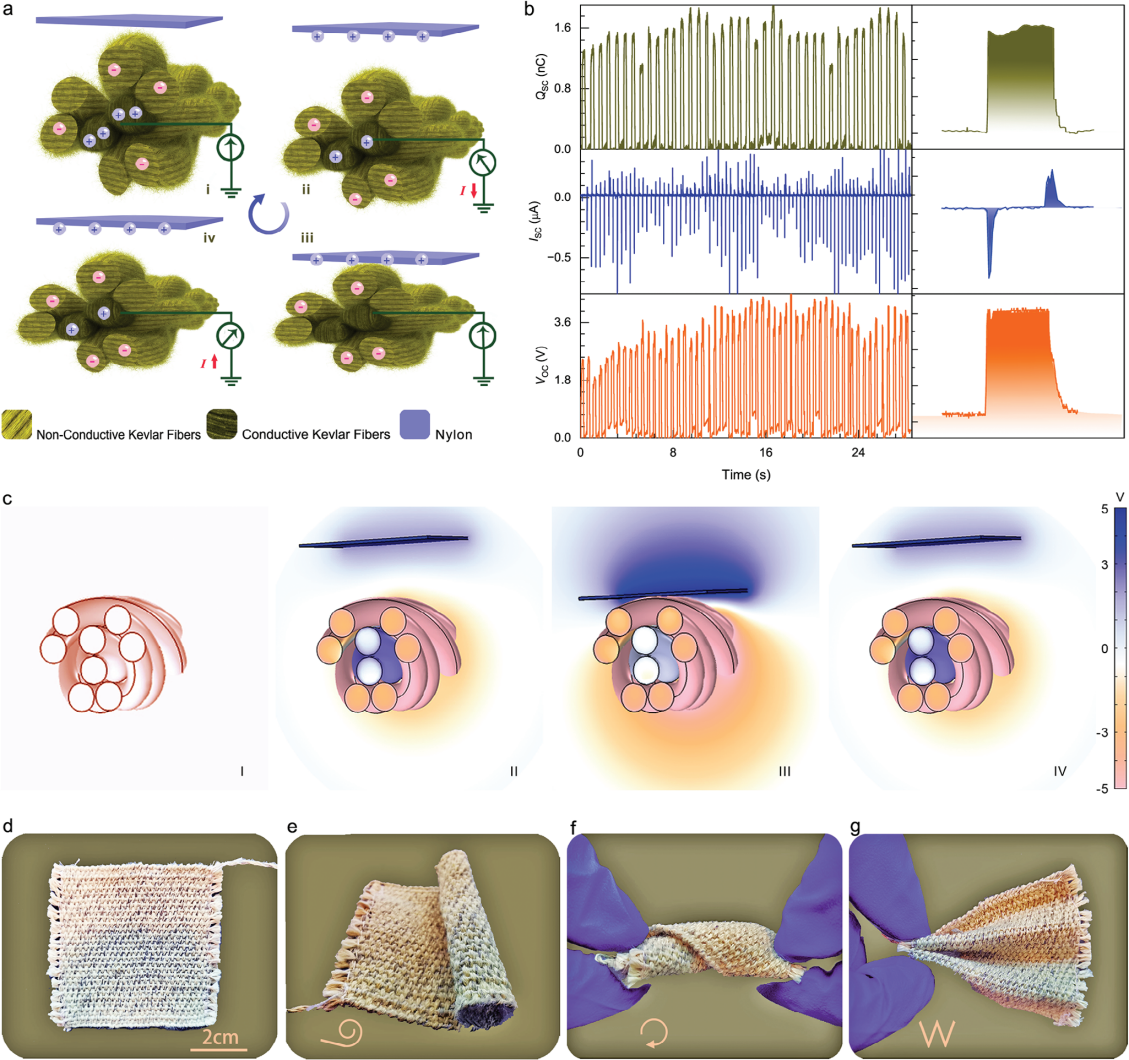
Principle of MTTK yarn and properties of SP‐ISFP. a) Working principle of MTTK yarn under one press and recovery working cycle. b) The *I*_SC_, *Q*_SC_, and *V*_OC_ under pressing working mode. c) Charge distribution of MTTK yarn in different states by finite‐element simulation. d) Optical photographs of the SP‐ISFP and e–g) its flexibility during twisted, rolled, and bent.

The MTTK yarn will operate in single‐electrode mode to meet the practical needs of wearing demand. In order to explain the working mechanism in this mode, nylon is used as a moving object. The working mechanism of MTTK yarn is shown in **Figure** **2**a. In the initial state (i), both the conductive Kevlar fibers and the non‐conductive Kevlar fibers possess an equal amount of heterogeneous charge, thereby rendering the entire MTTK yarn electrically neutral. However, as the nylon comes into contact with the MTTK yarn, the non‐conductive Kevlar fibers gain negative triboelectric charges due to their higher affinity for capturing negative charges. Consequently, the nylon becomes positively charged, resulting in an increasing potential difference between the two surfaces. This potential difference triggers an instantaneous flow of electrons from the conductive Kevlar fibers electrode to the ground through the external circuit (ii). This transient flow of electrons persists until complete contact is established between the nylon and the MTTK yarn (iii). However, when the nylon starts to separate from the MTTK yarn, the repulsion force causes the electrons to be pushed back toward the conductive Kevlar fibers electrode. This flow of electrons is redirected from the ground and passes through the external load (iv). Through the repetitive contact‐separation movement between the moving dielectric object and the MTTK yarn, an alternating current (AC) is generated. In order to quantitatively evaluate the output performance of MTTK yarn, nylon gloves were used as moving media objects, the contact area of nylon gloves relative to the MTTK yarn is 0.9 cm^2^. Because Kevlar fiber has good hygroscopic properties, in order to maximize the effect of humidity on output performance, all indoor tests provide 47% ambient humidity (normal ambient humidity range is 40−60%). The measurement results of short‐circuit current (*I*_SC_), transferred charge (*Q*_SC_), and open‐circuit voltage (*V*_OC_) are presented in Figure 2b. Additionally, COMSOL simulations depict the potential distribution in four different states, as shown in Figure 2c.

Using MTTK yarn as weft yarns, SP‐ISFP was prepared by the simplest plain weave process on a loom, as shown in Figure 2d. The actual photographs shown in Figure 2e–g demonstrate that the SP‐ISFP exhibits good variability and can be twisted, crimped, and bent, highlighting its excellent flexibility and adaptability. Air permeability is a critical parameter in the context of smart textiles. In order to evaluate this property, we conducted a comparative analysis between the SP‐ISFP and other commercially available stab‐resistant suits. As illustrated in Figure S3 (Supporting Information), the SP‐ISFP exhibits an air permeability of 420 mm s^−1^ under a pressure of 1 kPa, while commercial Kevlar fabrics and 2 mm PE lack breathability. Additionally, 1 mm PE demonstrates satisfactory air permeability under higher pressure but falls short when compared to the SP‐ISFP under pressures below 0.72 kPa. This outcome clearly demonstrates that the SP‐ISFP outperforms other commercially available stab suits in terms of air permeability, thereby ensuring its suitability for operational conditions.

**Figure 3:**
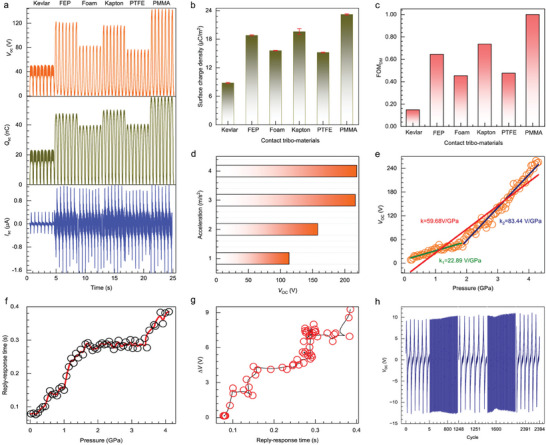
Electrical characterizations of SP‐ISFP. a) *V*_OC_, *Q*_SC_, and *I*_SC_ inspection signals of six different contact dielectric materials. b) Transfer charge density of different dielectric materials. c) FOM_DM_ of six dielectric materials. d) *V*_OC_ of SP‐ISFP at different accelerations. e,f) Linearity, and sensitivity of SP‐ISFP under different pressures. g) The relationship between sensitivity and accuracy. h) The *V*_OC_ of the SP‐ISFP under 2400 impact cycles.

Given the dependence of the surface charge density of the SP‐ISFP on the type of dielectric material in contact. In order to investigate this phenomenon in the context of the bulletproof vest structure, six different dielectric materials were individually tested for their contact with the SP‐ISFP under identical force conditions. The dielectric materials subjected to our testing included commercial Kevlar fabric, fluorinated ethylene propylene (FEP) film, soft foam, Kapton film, polytetrafluoroethylene (PTFE) film, and acrylic sheet (PMMA), the electrical output test model for varying the dielectric material is shown in Figure S4 (Supporting Information). The triboelectric order, as determined by our measurements, is as follows (most negative): PMMA‐ Kapton‐FEP‐PTFE‐soft foam‐Kevlar fabric (most positive), the corresponding test results are presented in **Figure** **3**a. The transfer charge density is calculated based on the measured transfer charge, as shown in Figure 3b. The triboelectric performance of each material can be standardly quantified using the triboelectric surface charge density with respect to a certain material. For example, the triboelectric performance of FEP can be quantified as δ_FEP/Kevlar_ = 1.705 C m^−2^ with respect to Kevlar. In this work, PMMA is considered the most triboelectric negative material; Kevlar should be about in the middle position of the triboelectric series. Therefore, we can consider the surface charge densities while contacting PMMA with Kevlar as the reference triboelectric charge densities. Then, the normalized triboelectric charge density δ_N_ and dimensionless material FOM (FOM_DM_) for triboelectrification (with respect to the charge density of PMMA contacting with Kevlar) can be defined as^[^ ^26^ ^]^:

(1) $$FOM_{DM}=\left(\delta^{2}\right)=\frac{\delta_{Material/Kevlar}^{2}}{\delta_{PMMA/Kevlar}^{2}}$$

Then the normalized triboelectric charge densities and FOM_DM_ of measured materials are listed in Figure 3c. For a certain material, if δ_N_ _<_ 0, then the material is more positive than the reference Kevlar; If 0 < δ_N_ < 1, then this material is more negative than the reference Kevlar and more positive than PMMA; If δ_N_ > 1, then this material is more negative than PMMA.

Based on the aforementioned calculation results, it has been determined that the dielectric material in question is PMMA. The morphological characteristics of impact damage vary greatly depending on the impact velocity; therefore, we tested the *I*_SC_, *Q*_SC_, and *V*_OC_ of the SP‐ISFP at different accelerations, at the same distance, the faster the acceleration, the greater the impact velocity on the SP‐ISFP, and the electrical output results are shown in Figure 3d and Figure S5 (Supporting Information). Sensing is a critical element in impact‐resistant smart sensors, and the linearity, accuracy, and sensitivity of the pressure response exhibited by the SP‐ISFP under high‐impact conditions are of utmost importance. To evaluate the output response properties of the SP‐ISFP, hammer impacts are utilized as the moving object, and the SP‐ISFP operates in single electrode mode. Thereafter, the output of the SP‐ISFP is tested at different pressures to evaluate its output response properties, Figure 3e displays the *V*_OC_ response of SP‐ISFP under different pressures. This Figure shows that the *V*_OC_ increased as the applied pressure increased from 0 to 4.2 GPa. This higher *V*_OC_ output is caused by the formation of larger contact areas due to increased pressure. Furthermore, the response curve of pressure exhibits two distinct regions. In the low‐pressure region (0–2 GPa), the SP‐ISFP exhibits a well‐behaved linear response with a pressure sensitivity of 22.89 V GPa^−1^; However, in the high‐pressure region (>2 GPa), the pressure sensitivity is 83.44 V GPa^−1^. With the increase of pressure, SP‐ISFPs transition from point contact to surface contact, and the contact area gradually increases, especially ≈2 GPa. For flexible fabrics, with the increase of pressure, the contact area will not increase linearly with the increase of pressure, especially after 2 GPa, with the increase of pressure, the shape change has reached the maximum, and further increasing the pressure will only increase the effective contact area. The maximum *V*_OC_ is 265 V at 4 GPa. Movie S2 (Supporting Information) showcases the SP‐ISFP's ability to maintain normal function even when subjected to an impact from a 4 GPa hammer. However, noticeable damage in the form of a pit and long crack can be observed on the lower planks. The response time of the sensing unit, which is crucial for real‐time monitoring, was measured and depicted in Figure 3f. The push‐release cycle demonstrates a response time of less than 0.4 s under 4 GPa, ensuring prompt feedback. Figure S6 (Supporting Information) illustrates the accuracy of the voltage output of the SP‐ISFP at various pressures. It can be observed that the error tends to increase as the pressure increases. However, even at the highest pressure of 4 GPa, the maximum error does not exceed 10 V. In addition, the relationship between the response time and the error is also calculated. It can be seen from Figure 3g that even when the response time is 0.4 s, the error of SP‐ISFP is not more than 10 V, indicating that SP‐ISFP has excellent accuracy. One of the unique advantages of triboelectric technology is that triboelectric materials can convert impact energy into electrical energy, thus reducing the probability of the wearer being exposed to external injuries. so, the energy conversion efficiency of SP‐ISFP at different speeds is calculated when the impact force is 100 N, and the energy conversion efficiency is 2.4% when the speed is 0.3 m s^−1^, as shown in Table S1 (Supporting Information). Furthermore, durability is a crucial parameter for wearable smart fabric. In Figure 3h, it can be observed that the *V*_OC_ remains stable after continuous operation for 2400 cycles, substantiating the superior stability of our SP‐ISFP.

If the SP‐ISFP connects with capacitors or batteries, the generated electricity can be stored. This accumulated and stored charge can be utilized to sustainably power wearable electronics. To study the ability of the SP‐ISFP to store charge, its charging curve under different pressures and capacitors is plotted, the charging speed decreases as the capacitance increases, and the charging speed increases with the increase of the applied force, the specific charging curve is shown Figure S7 (Supporting Information). To assess the effective output performance of the SP‐ISFP, we conducted tests under various resistances, as the external resistances increased, the voltage in the SP‐ISFP also increased. However, the instantaneous power exhibited an interesting trend, initially increasing and then decreasing. At an acceleration of 4 m s^−1^, the instantaneous power reached its peak at 0.17 mW when the load resistance was set to 140 MΩ. The relationship between voltage and instantaneous power with respect to the external resistance at different accelerations can be found in Figure S8 (Supporting Information).

**Figure 4:**
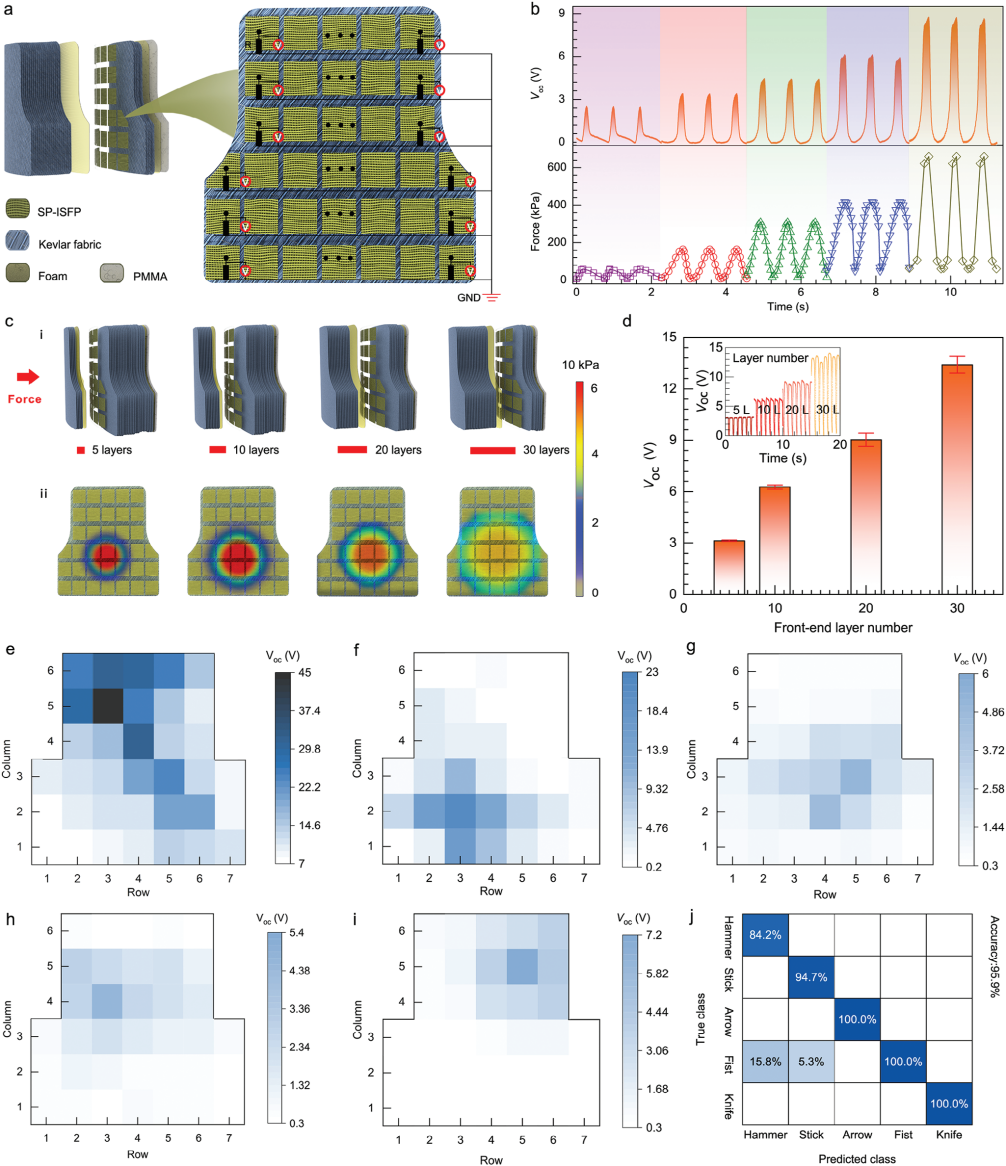
The array of 36 SP‐ISFPs performances and color mapping. a) Array position of 36 SP‐ISFPs in the bulletproof layer and working principle of the multi‐channel measurement system for a smart bulletproof vest. b) The synchronized diagram of the *V*_OC_ obtained by the smart bulletproof vest and the corresponding pressure measured by the commercial sensor. c‐i) Position model of 36 SP‐ISFPs arrays in a bulletproof vest. c‐ii) The stress distribution under different front‐end layer numbers is simulated by the finite element method. d) The influence of the number of front‐end layers on *V*_OC_ was studied. e–i) Change the type of impact force applied, and the output voltage corresponds to the rainbow color diagram. j) Confusion matrix of the prediction set (accuracy of 95.9%).

Integrated the array of 36 SP‐ISFPs into the bulletproof vest, the photo is shown in **Figure** **4**a, in which the manufacturing method, size, and spacing are all the same for every unit. All units are connected to the synchronized data acquisition card (PXIe‐4300, National Instruments) with an integrated signal conditioning system and coded with “Row” and “Column” for multi‐channel output voltage measurement. The applied pressure is controlled by a stepper motor program and the pressure is measured by a commercial manometer (resolution ≈1 mN), as can be seen from Figure 4b, when SP‐ISFP is placed in a bulletproof vest, there is still a linear relationship between output and pressure. A more detailed relationship between *V*_OC_ and pressure can be found in Figure S9 (Supporting Information), the linear fitting of the data yields a coefficient of determination (*R* ^2^) value of 0.98 and a slope of 0.0211.

Furthermore, we also observe that the position of the array of 36 SP‐ISFPs has an impact on its output. Figure 4c‐i illustrates the position of the array of 36 SP‐ISFPs, while Figure 4c‐ii demonstrates the stress situation of the array of 36 SP‐ISFPs simulated using the finite element method. We examine the effect of different numbers of layers at the front end of the array of 36 SP‐ISFPs under the same force area. Our results show that with an increased number of layers, the impact force becomes more dispersed, leading to a more accurate representation of the stressed areas of the body during impact. The change in the number of front‐end layers also affects the output signal of a single SP‐ISFP. The specific output signal is depicted in Figure 4d and Figure S10 (Supporting Information), showing that the electrical output increases as the number of front‐end layers increases. For instance, when there are only five layers at the front end, the measured output voltage of the SP‐ISFP is 3.6 V. However, when the number of front‐end layers reaches 30, the output voltage increases to 14.2 V, this increase can be attributed to changes in the stress distribution within the SP‐ISFP. This phenomenon is further supported by the results of finite element analysis, simulation is conducted with a smaller force area, and with an increase in the number of front layers, the stress area between the array of 36 SP‐ISFPs and the friction layer also enlarges. This can be visually observed in the stress analysis diagram presented in Figure S11 (Supporting Information).

In addition, the morphological characteristics of impact damage vary with the surface shape of the impactor and the impact site of the human body, Figure 4e‐i represents the impact of the stick, hammer, knife, arrow and fist on the bulletproof vest, and the array of 36 SP‐ISFPs displays the impact position and output size through the rainbow color image. Among them, the stick's electrical output is the largest, indicating that its impact is the most serious, and the rainbow color image also shows the location and scope of its impact. Figure S12 (Supporting Information) assisted in simulating the corresponding force analysis under the impact of different impactors. The rainbow color image of the array of 36 SP‐ISFPs can easily determine the impact position of the user and the surface shape of the impactor, so as to provide timely rescue assistance to the user. To accurately assess the type of impactor and impact site, we analyze and identify multidimensional and vast data by using machine learning. Each impactor was repeated fifty times, and the output signals for most weapons exhibited distinct waveforms and peaks, Figures S13–S17 (Supporting Information) show the original waveforms of the five impactors acting fifty times. Figure 4j provides a prediction confusion matrix for the output signals of the five types of impactors based on prototype learning, the total recognition accuracy of the current model is 95.9%, and the probability of “Hammer” being recognized as “Fist” is 15.8%. The confusion matrix of the training model is shown in Figure S18 (Supporting Information).

**Figure 5:**
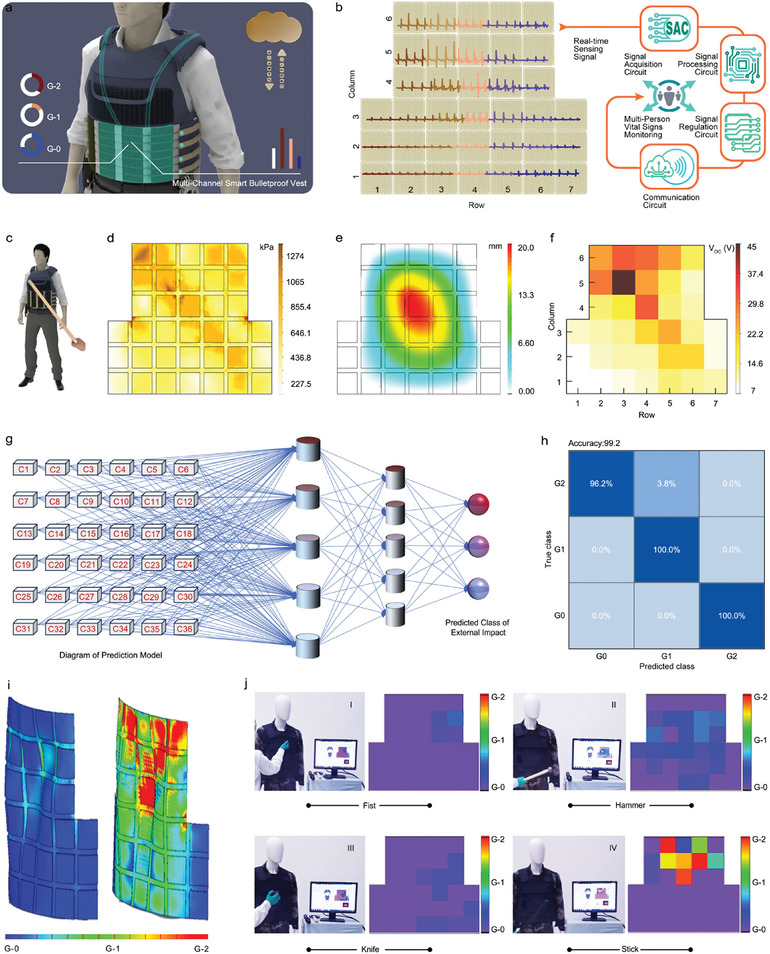
Real‐time monitoring of body impact by using a multi‐channel smart bulletproof vest and grading assessment of impact. a) Application diagram about multi‐channel smart bulletproof vest. b) Operational scheme diagram of the the multi‐channel smart bulletproof vest. c) Statement on impact location of a multi‐channel smart bulletproof vest. d) Stress distribution of multi‐channel smart bulletproof vest by finite‐element simulation. e) Displacement changes of multi‐channel smart bulletproof vest by finite‐element simulation. f) the output voltage corresponds to the rainbow color diagram. g) Conceptual diagram of CNN for impact grade classification. h) Confusion matrix of the prediction set (accuracy of 99.2%). i) 3D cloud map distribution at G2 of body impact. j) Demonstration of the LabVIEW interface, screenshot showing the real‐time statistical result of the self‐powered system.

As mentioned above, the array of 36 SP‐ISFPs can well identify the force and location of the impact, we propose a multi‐channel smart bulletproof vest, sensing and information interaction system that eliminates the need for integrated microprocessors, batteries, Bluetooth modules and other electronic components for human in high impact conditions, as depicted in **Figure** **5**a. The scheme diagram of the multi‐channel smart bulletproof vest is shown in Figure 5b. The signal produced by the array of 36 SP‐ISFPs goes to the signal acquisition when the user is affected. The signal processing circuit transforms the electrical signal into a digital signal that is embedded in the microcontroller in channel order as the analog signal passes through it. The communication circuit transmits the converted digital signal to the display screen in less than 1 s, used for multi‐person vital signs monitoring.

When the user is attacked as shown in Figure 5c, different parts of the multi‐channel smart bulletproof vest receive different degrees of stretching and compression, the array of 36 SP‐ISFPs resulting in different signal values, and the rainbow plot in Figure 5f shows different impact locations and impact sizes. At the same time, through finite element analysis, we can simulate the distribution of stress and displacement caused by applied force, as shown in Figure 5d,e. According to Merkle et al.^[^ ^27^ ^]^ and bulletproof vest standards, for effective protection, the deformation of the back of the vest should not exceed 44 mm. If this limit is exceeded, the body will suffer serious damage. It can be seen from the displacement distribution that the maximum deformation produced by the back of the vest is 20 mm. From the above analysis, we can comprehensively evaluate the user's vital signs from the aspects of electrical signal, stress, and displacement to ensure the accuracy of the conclusion. A more detailed output voltage signal can be found in Figure S19 (Supporting Information).

For precise assessment of body impact, we analyze and identify multidimensional and vast data by using a convolutional neural network (CNN) (Figure 5g). This method is one of the most mature and simple machine learning algorithms. The data generated by the array of 36 SP‐ISFPs during the impact process is aggregated by data acquisition card, we're grading injuries on a scale of 0–2 (G0 to G2). Before modeling operations, we need to clean and extract features from the massive amount of data. Different impactors are subjected to repeat testing fifty times under different impacts, resulting in varying peak magnitudes in the output signals for each impact. Figure S20 (Supporting Information) gives the peak area of G0 to G2. The peak value greater than 16.8 V is judged as G2, the peak value within 16.79–5.22 V is judged as G1, and the peak value less than 5.22 V is judged as G0. Figure 5h provides the prediction confusion matrix for the three levels (G0 to G2) based on machine learning, the accuracy achieved is 99.2%. The detailed confusion matrix of the training model based on the impact level is shown in Figure S21 (Supporting Information).

With objective and reliable evaluation standards, the array of 36 SP‐ISFPs and the associated machine‐learning algorithm allows for real‐time wireless visual monitoring of impacts from impactors like fists, hammers, clubs, and knives. The electrical signals generated synchronously by the array of 36 SP‐ISFPs and the impactor impact are collected by the multi‐channel data acquisition card, processed, and displayed by the software based on LabVIEW, as shown in Figure 5j. This LabVIEW interface encompasses an impact distribution map, an impactor identification diagram, and an impact feedback alarm diagram, this interface can reconstruct the pressure distribution. To demonstrate its functionality, a baton was utilized to impact the same positions on the smart bulletproof vest five times, each time the impact strength was changed, a corresponding update was generated in the impact distribution map, and the feedback of G0, G1, and G2 on different forces (Movie S3, Supporting Information). This is a simple and convenient way to judge the user's physical state, whether to continue fighting or provide rescue. In addition to the aforementioned features, the multi‐channel smart bulletproof vest is capable of determining the type of impactor used in an attack based on the force size and force position. It can assess the user's physical condition and provide timely rescue if needed. Movie S4 (Supporting Information) showcases a test conducted to evaluate impactor type identification and alarm notification using the multi‐channel smart bulletproof vest. When the wearer is attacked with a fist, a corresponding image of a fist will be displayed in the impactor identification diagram. The emojis representing “OK” (G0), “sad” (G1), and “crying” (G2) will be generated based on the strength of the impact. Furthermore, the alarm light will flash when the emojis indicating “sad” (G1), and “crying” (G2) are displayed. By analyzing the impact results, the user's physical condition can be understood in a timely manner, thus providing further guidance for the actions of the rescue team and enabling the formulation of more effective rescue plans. This prototype of the multi‐channel smart bulletproof vest demonstrates the potential to create a new type of protective equipment for both combat and rescue personnel. Figure 5i shows the 3D rainbow color maps of body impact representing injured grades and locations. The results demonstrated that the multi‐channel smart bulletproof vest can quickly pinpoint the area of injury and offer precise and intuitive advice. The advantage lies in the potential reduction of incidents that delay diagnosis and treatment.

## 3 Conclusion

In summary, we have developed an MTTK yarn composed of conductive and non‐conductive Kevlar fibers. This yarn exhibits a high tensile strength of 372 MPa and possesses good electrical conductivity of 6.95 × 10^−3^ s m^−1^. The MTTK yarn is used to design an SP‐ISFP, which demonstrates excellent impact resistance, functioning properly even under 4 GPa impact. The SP‐ISFP achieves a maximum *V*_OC_ of 252 V and generates an output power of 0.17 mW. This power output is sufficient to charge capacitors and effectively power commercial electronics. The CNN as a decision model was built with the assistance of machine learning methods and the severity indices (0 to 2) of body impact were assessed with an accuracy of 99.2% by algorithm optimization. A multi‐channel smart bulletproof vest was created to provide prediagnostic references in practical applications, for example, in the case of user injury, medical personnel can judge whether to provide assistance through the conclusion displayed in the terminal. This demonstrates the potential of our work in providing a promising safety net for individuals operating in high‐risk environments, particularly in the context of wearable protective clothing.

## 4 Experimental Section

### Fabrication of the Conductive Kevlar fibers

Conductive Kevlar fibers were prepared by repeatedly soaking non‐conductive Kevlar fibers (nominal diameter 0.66 mm) in a silver nanowire solution (a mixture of silver nanowires and ethanol with a diameter of 30 nm). The silver nanowire solution could be obtained from Xianfeng Nano Co., LTD. The non‐conductive Kevlar fibers (Yixing city siweiqi carbon fiber products stored on Taobao) were soaked at room temperature for 2 h in 5 mg mL^−1^ silver nanowire solution and then dried repeat three times.

### Fabrication of the Multistrand Twisted Triboelectric Kevlar (MTTK) Yarn

Conductive Kevlar fibers (nominal diameter: 0.66 mm, resistance <1.44 × 10^2^ Ω m,) was chosen as the electrode. The MTTK yarn was prepared on a high‐speed rope‐braiding machine (Figure S1, Supporting Information). First, conductive Kevlar yarns were wound on the bobbins, which were fixed on the spindles. The spindle was fastened to the directly behind the motor machine bed, the conductive Kevlar fibers pass through the motor and the machine bed and do not rotate with the rotation of the motor. Second, non‐conductive Kevlar fibers were wound on the bobbins, which were fixed on the spindles. The spindle was fastened in the motor machine bed, the machine bed rotated with the motor, and the non‐conductive Kevlar fibers were wrapped around the conductive Kevlar fibers in the middle as the machine rotated. The continuous supply of yarns was achieved by the rotation of the bobbin on the spindle.

### Fabrication of the Self‐Powered Impact Sensing Fabric Patch (SP‐ISFP)

A semi‐automatic weaving machine was used for weaving SP‐ISFP. First, the non‐conductive Kevlar fibers were arranged on the loom to prepare as warp yarn; Second, the conductive Kevlar fibers were wrapped around the shuttle as filling yarns; finally, the shuttle interweaved in the warp and woven SP‐ISFP.

### Characterization and Measurements

The morphology of the conductive Kevlar fiber was analyzed by scanning electron microscopy (SU8020, Hitachi Group, Japan). Mechanical properties of the MTTK yarn were measured with an electronic universal testing machine (model no. E3000, from Instron Corporation). For the measurement of the electrical output capability of the SP‐ISFP, external forces were applied by a mechanical motor (homemade equipment), which corresponded to the stretching and compressing operations, respectively. The open‐circuit voltage, short‐circuit current and transferred charge of the MTTK yarn and SP‐ISFPs were recorded with an electrometer (Keithley 6514).

## Conflict of Interest

The authors declare no conflict of interest.

## Author Contributions

F.X., X.G., and J.W. contributed equally to this work. F.X. wrote the original draft and contributed to methodology. X.G. performed data curation, validation, and methodology. J.W. performed investigation and formal analysis. H.L. and H.L. performed visualization. B.C. did supervision, acquired resources, wrote the original draft, and reviewed and edited the final manuscript. Z.L.W. performed conceptualization, supervision, resources acquisition, wrote the original draft and reviewed and edited the final manuscript.

## Supporting information

Supporting Information

Supplemental Movie 1

Supplemental Movie 2

Supplemental Movie 3

Supplemental Movie 4

## Data Availability

The data that support the findings of this study are available in the supplementary material of this article.
