# Supplementary material for: Multistrand Twisted Triboelectric Kevlar Yarns for Harvesting High Impact Energy, Body Injury Location and Levels Evaluation
Source: Adv Sci (Weinh). 2024 Mar 15;11(21):2401076. doi: 10.1002/advs.202401076 (PMC11151034; doi:10.1002/advs.202401076)
Supplement: Supplementary file 1 — Supporting Information [file ADVS-11-2401076-s004.pdf]

ADVANCED SCIENCE
Open Access

# Supporting Information

for *Adv. Sci.*, DOI 10.1002/advs.202401076

Multistrand Twisted Triboelectric Kevlar Yarns for Harvesting High Impact Energy, Body Injury Location and Levels Evaluation

*Fangjing Xing, Xiaobo Gao, Jing Wen, Hao Li, Hui Liu, Zhong Lin Wang* and Baodong Chen**

# Supporting Information

## Multistrand twisted triboelectric Kevlar yarns for harvesting high impact energy, body injury location and levels evaluation

*Fangjing Xing[#], Xiaobo Gao[#], Jing Wen[#], Hao Li, Hui Liu, Zhong Lin Wang*, and Baodong Chen**

F. Xing, X. Gao, J. Wen, H. Li, Li, Z. L. Wang, B. Chen
Beijing Institute of Nanoenergy and Nanosystems, Chinese Academy of Sciences, Beijing 101400, P. R. China
E-mail: zlwang@gatech.edu; chenbaodong@binn.cas.cn
F. Xing, J. Wen, H. Li, Li, Z. L. Wang, B. Chen
School of Nanoscience and Engineering, University of Chinese Academy of Sciences, Beijing 100049, P. R. China
X. Gao
School of Materials Science and Engineering, Inner Mongolia University of Technology, Hohhot 010051, P. R. China
H. Liu
Changchun University of Chinese Medicine, Jilin 130117, P. R. China
Z. L. Wang
Georgia Institute of Technology, Atlanta, GA30332, USA

[#] *These authors contributed equally: F. Xing, X. Gao and J. Wen*

** Co-corresponding authors: Z. L. Wang, and B. Chen*

*e-mail: zlwang@gatech.edu (Z. L. Wang); chenbaodong@binn.cas.cn (B. Chen)*

**List**

**Table S1.** Energy conversion efficiency of a SP-ISFP.

**Figure S1.** The process of manufacturing MTTK yarn.

**Figure S2.** Photographs showing the flexibility of MTTK yarn.

**Figure S3.** Comparison of air permeability between the SP-ISFP and several commercial stab resistant suits.

**Figure S4.** Contact models of six dielectric materials and their effects on the output of SP-ISFP

**Figure S5.** Comparison of electrical output of SP-ISFP under different accelerations.

**Figure S6.** Accuracy of SP-ISFP under different pressures.

**Figure S7.** Charging capacity of the SP-ISFP at different the external forces.

**Figure S8.** Dependence of the Resistance and peak power at different accelerations.

**Figure S9.** Electrical output performances of SP-ISFP at different the external forces.

**Figure S10.** The interdependence between the number of front-end layers and the output electrical properties of SP-ISFP**.**

**Figure S11.** Simulation of the relationship between the number of layers at the front-end of a single SP-ISFP and the stress.

**Figure S12.** Pressure distribution of the array of 36 SP-ISFPs, predicted by finite element analysis via SolidWorks software.

**Figure S13.** The original waveform diagram of the stick acting fifty times.

**Figure S14.** The original waveform diagram of the hammer acting fifty times.

**Figure S15.** The original waveform diagram of the arrow acting fifty times.

**Figure S16.** The original waveform diagram of the fist acting fifty times.

**Figure S17.** The original waveform diagram of the knife acting fifty times.

**Figure S18.** Training set confusion matrix for weapon identification (accuracy of 98.7%).

**Figure S19.** Detail of SP-ISTPs voltage signal at 9 main force positions.

**Figure S20.** Impact grade prediction waveform diagram.

**Figure S21.** Training set confusion matrix for impact grade judgment (accuracy of 98.2%).

**Movie S1.** The process of manufacturing MTTK yarn.

**Movie S2.** The SP-ISFP can withstand high impact and output electrical signals.

**Movie S3.** The multi-channel smart bulletproof vest can determine the impact level and provide alarm services.

**Movie S4.** Demonstration of the multi-channel smart bulletproof vest.

Illustrative calculation of energy conversion efficiency

One of the unique advantages of triboelectric technology in bulletproof vest is that triboelectric materials can convert impact energy into electrical energy, thus reducing the probability of the wearer being exposed to external injuries. Since bulletproof vest is mostly converted to electricity from the impactor's kinetic energy on impact. Therefore, we tested the energy conversion efficiency of a SP-ISFP at different speeds. The mass of the object is 100g and the area of a SP-ISFP is $2.5\times10^{-3}m^2$. The energy conversion efficiency was calculated using the formula shown in below, as shown in Table S1 below.

Where m is the mass of the impactor, v is the instantaneous velocity when the impactor hits the SP-ISFP. U is the open-circuit voltage and I is the short-circuit current.

$$\eta = \frac{E_{elec}}{E_k} = \frac{\int_0^t UIdt}{\frac{1}{2}\mathrm{m}v^2}$$

**Table S1 Energy conversion efficiency of a SP-ISFP.**

| Speed (m/s) | Mass (Kg) | $E_{elec}$ | $E_k$ | η (%) |
|---|---|---|---|---|
| 0.30 | 0.1 | 0.00013 | 0.0054 | 2.4 |
| 0.54 | 0.1 | 0.00020 | 0.0146 | 1.3 |
| 0.56 | 0.1 | 0.00030 | 0.0157 | 1.9 |
| 0.57 | 0.1 | 0.00040 | 0.0162 | 2.4 |

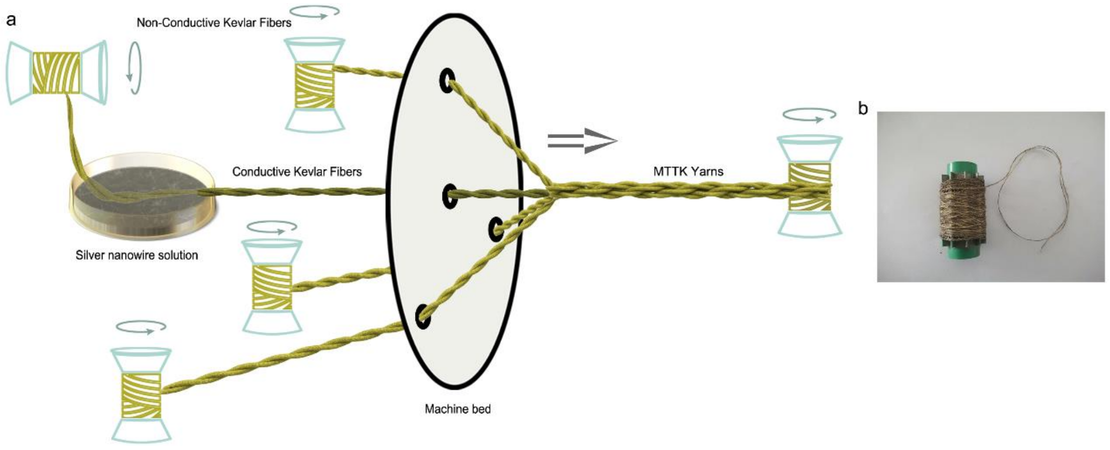


**Figure S1. The process of manufacturing MTTK yarn.** a) The soak, dry and twist process for manufacturing MTTK yarn. b) Optical photo of conductive Kevlar fibers.

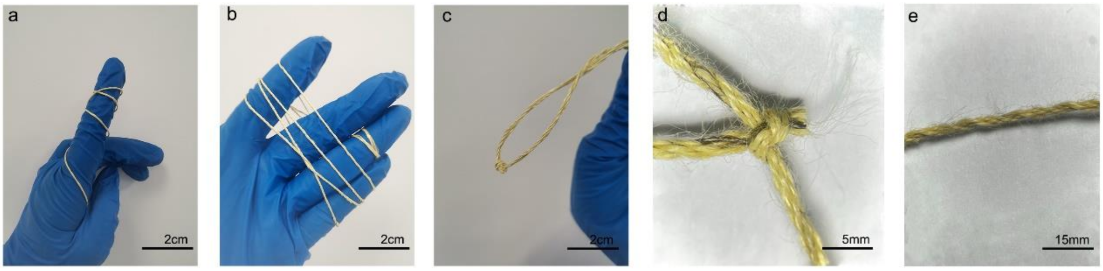


**Figure S2. Photographs showing the flexibility of MTTK yarn.** a, b) Entanglement, c, d) knotting and e microscopic magnification

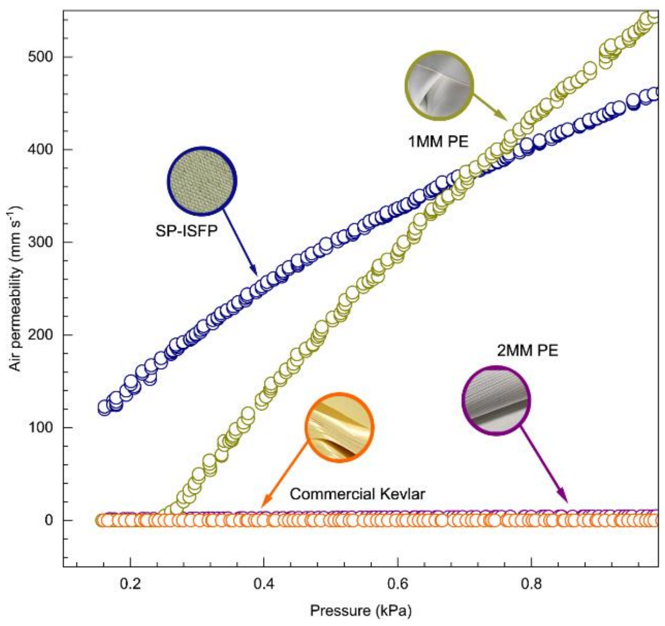


**Figure S3. Comparison of air permeability between the SP-ISFP and several commercial stab resistant suits.**

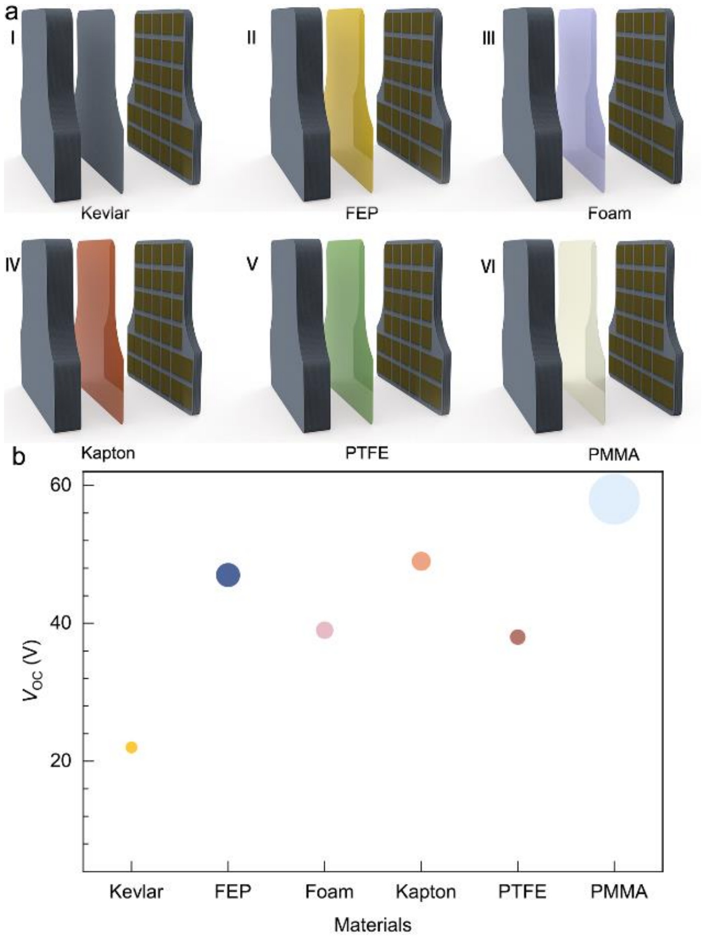


**Figure. S4. Contact models of six dielectric materials and their effects on the output of SP-ISFP.** a) I. Kevlar and SP-ISFP. II. FEP and SP-ISFP. III Foam and SP-ISFP. IV. Kapton and SP-ISFP. V. PTFE and triboelectric SP-ISFP. VI. PMMA and SP-ISFP. b) Comparison of electrical output signals of six dielectric materials and SP-ISFP.

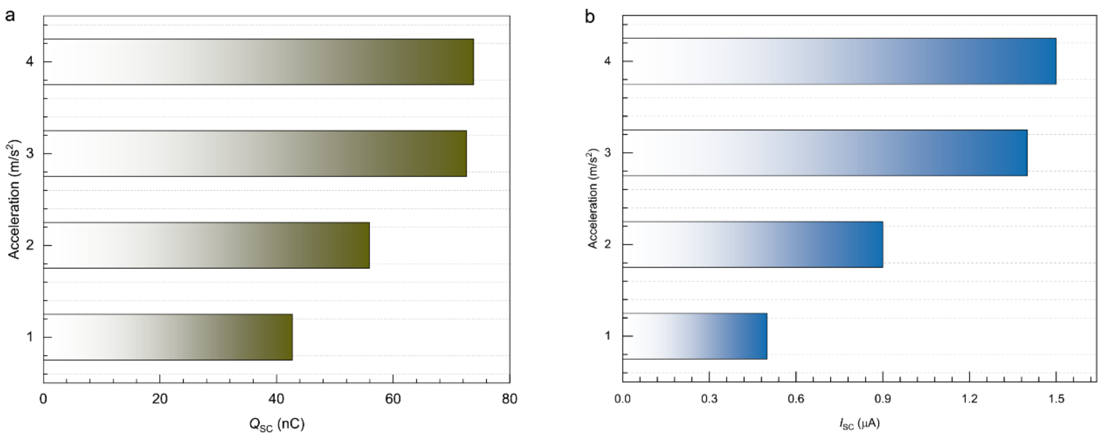


**Figure S5. Comparison of electrical output of SP-ISFP under different accelerations.** a) $I_{SC}$ of SP-ISFP under different accelerations. b) $Q_{SC}$ of SP-ISFP under different accelerations.

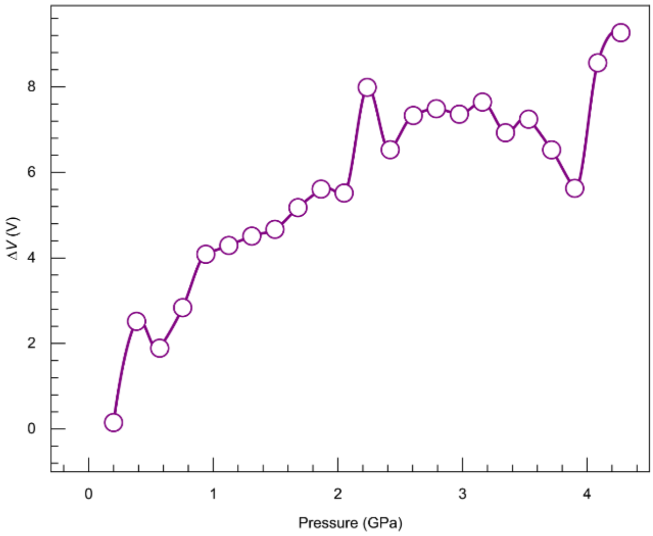


**Figure S6. Accuracy of SP-ISFP under different pressures.**

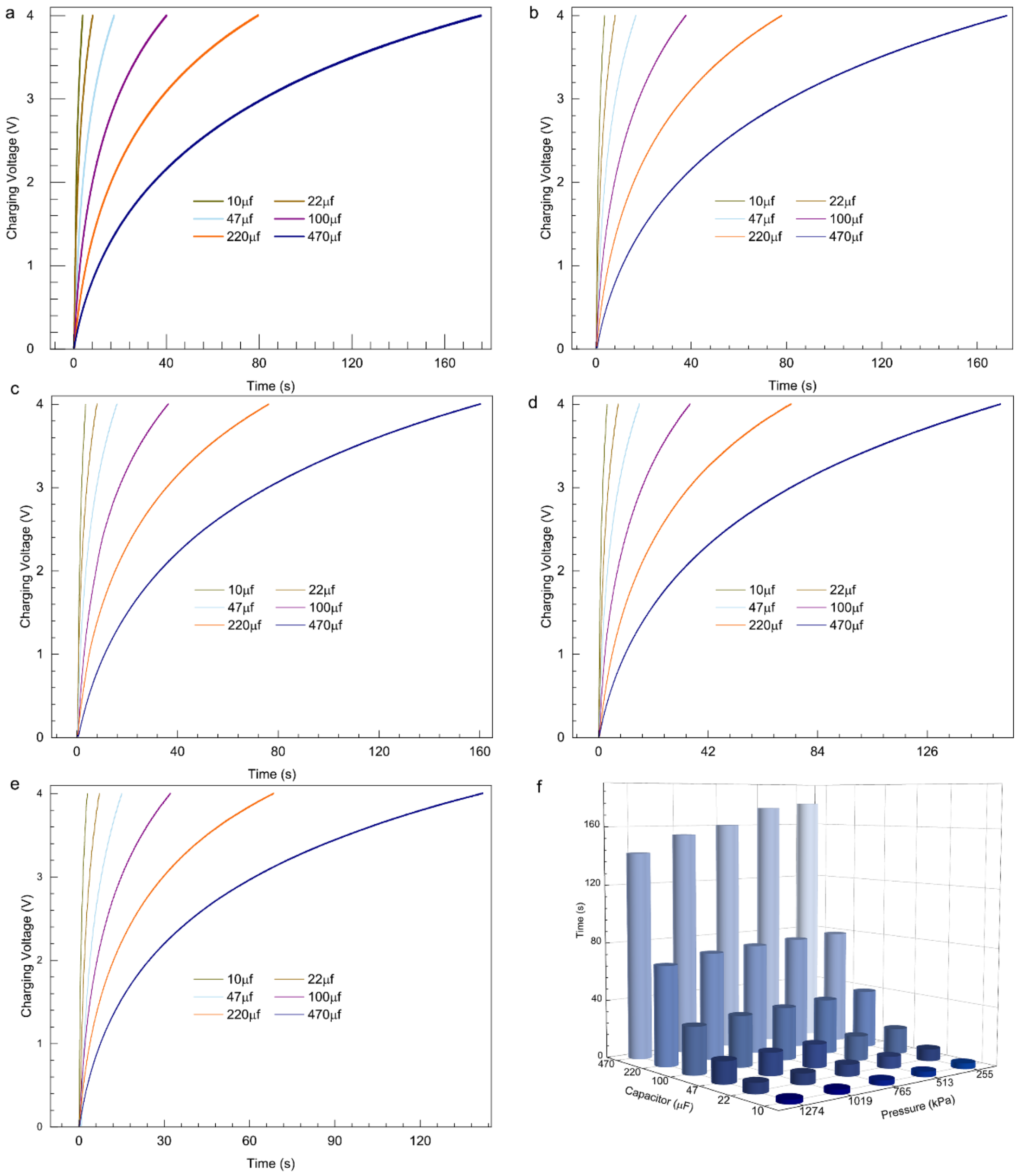


**Figure S7. Charging capacity of the SP-ISFP at different the external forces.** a) Charging voltage of the different capacitances (10 μF, 22 μF, 47 μF, and 100 μF) at the force of 225 kPa. b) Charging voltage of the different capacitances (10 μF, 22 μF, 47 μF, and 100 μF) at the force of 573 kPa. c) Charging voltage of the different capacitances (10 μF, 22 μF, 47 μF, and 100 μF) at the force of 769 kPa. d) Charging voltage of the different capacitances (10 μF, 22 μF, 47 μF, and 100 μF) at the force of 1019 kPa. e) Charging voltage of the different capacitances (10 μF, 22 μF, 47 μF, and 100 μF) at the force of 1274 kPa. f) Summary diagram of charging capacity of SP-ISFP

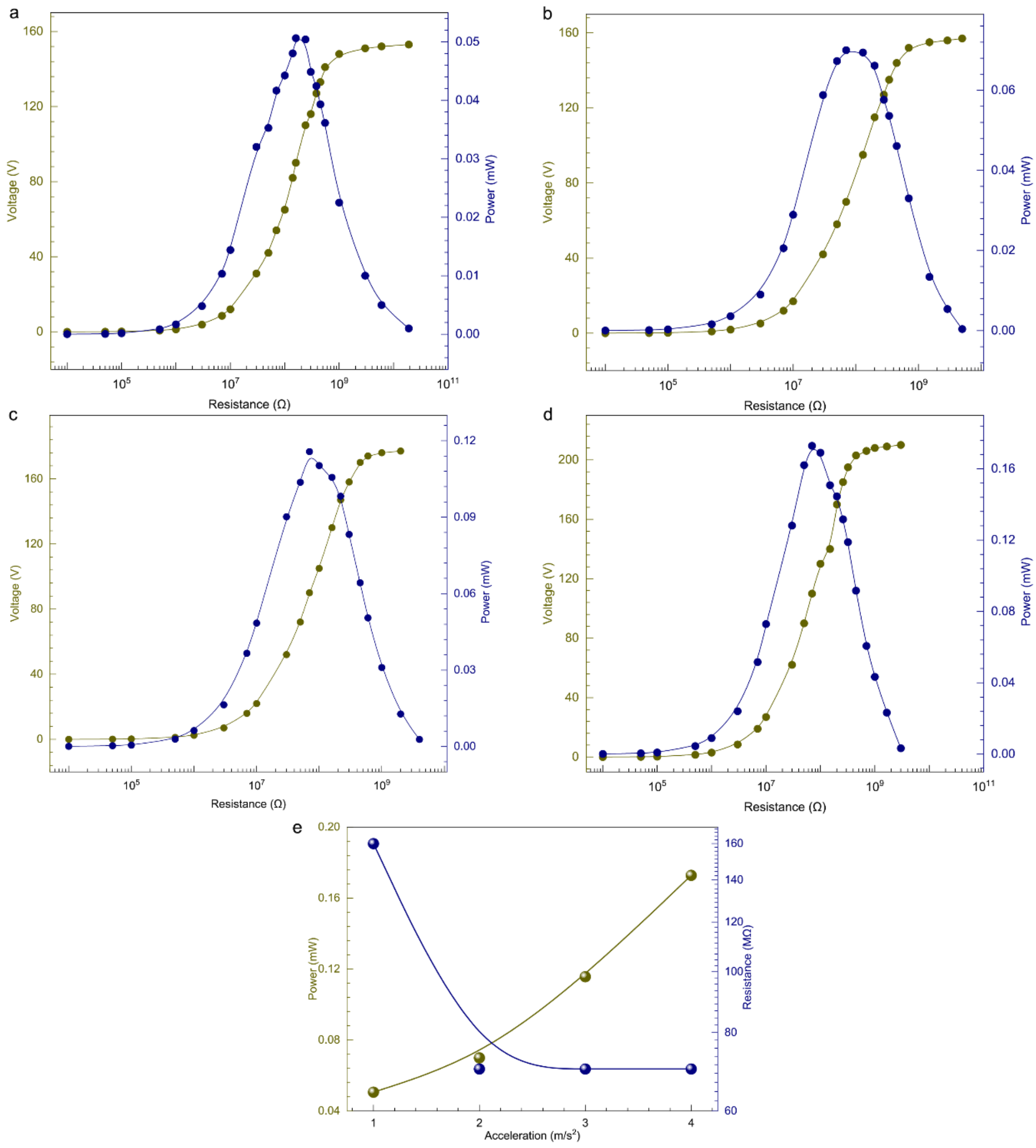


**Figure S8. Dependence of the Resistance and peak power at different accelerations.** a) The peak power corresponding to the voltage at 1 m/s$^2$ acceleration. b) The peak power corresponding to the voltage at 2 m/s$^2$ acceleration. c) The peak power corresponding to the voltage at 3 m/s$^2$ acceleration. d) The peak power corresponding to the voltage at 4 m/s$^2$ acceleration. e) Summary diagram of the relationship between external resistance and peak power.

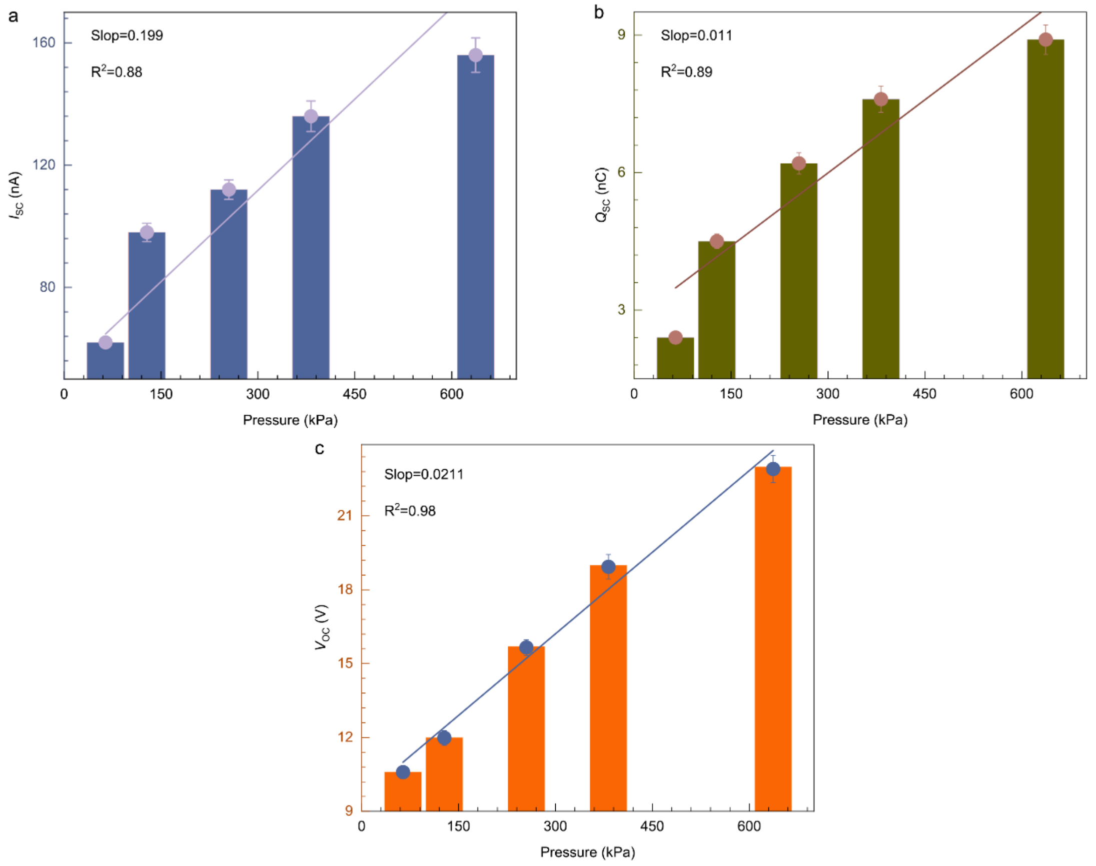


**Figure S9. Electrical output performances of SP-ISFP at different the external forces.** a) $I_{SC}$ of the SP-ISFP with the external forces of 125 kPa to 720 kPa. b) $Q_{SC}$ of the SP-ISFP with the external forces of 125 kPa to 720 kPa. c) $V_{OC}$ of the SP-ISFP with the external forces of 125 kPa to 720 kPa.

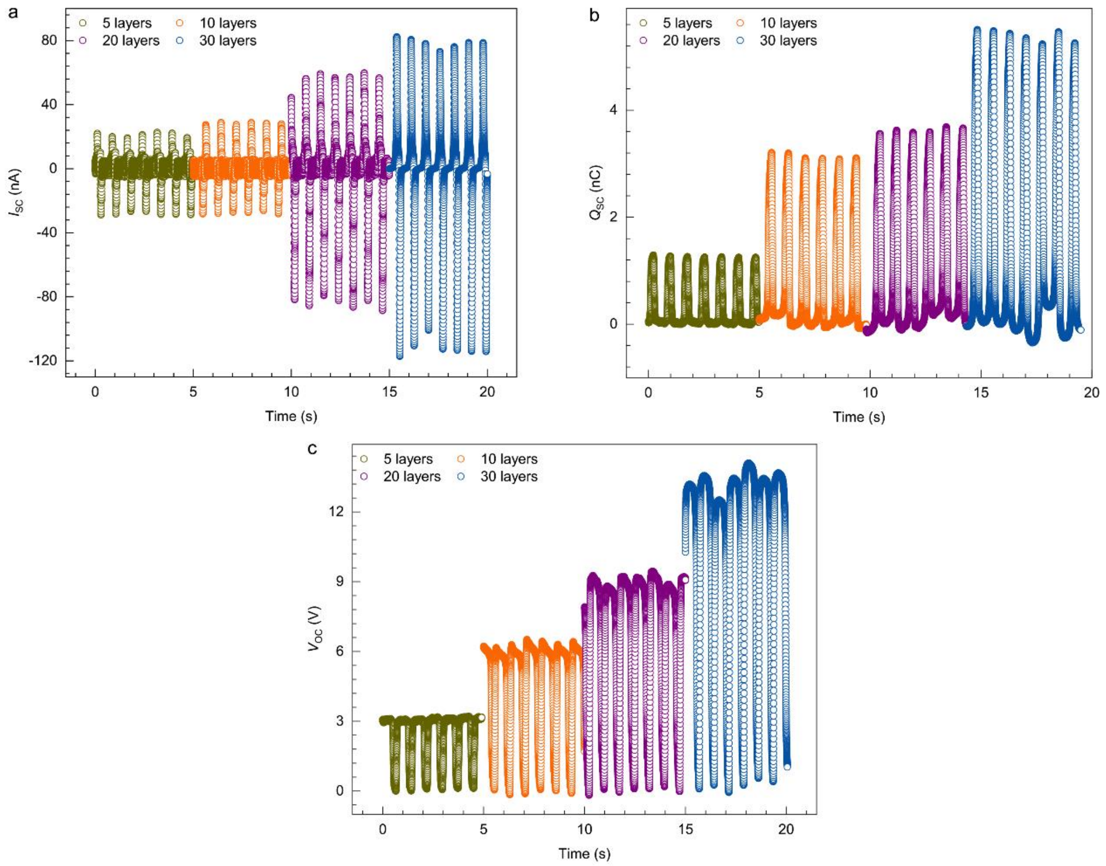


**Figure S10. The interdependence between the number of front-end layers and the output electrical properties of SP-ISFP.** a) $I_{SC}$ when the number of front-end layers is 5, 10, 20, and 30. b) $Q_{SC}$ when the number of front-end layers is 5, 10, 20, and 30. c) $V_{OC}$ when the number of front-end layers is 5, 10, 20, and 30.

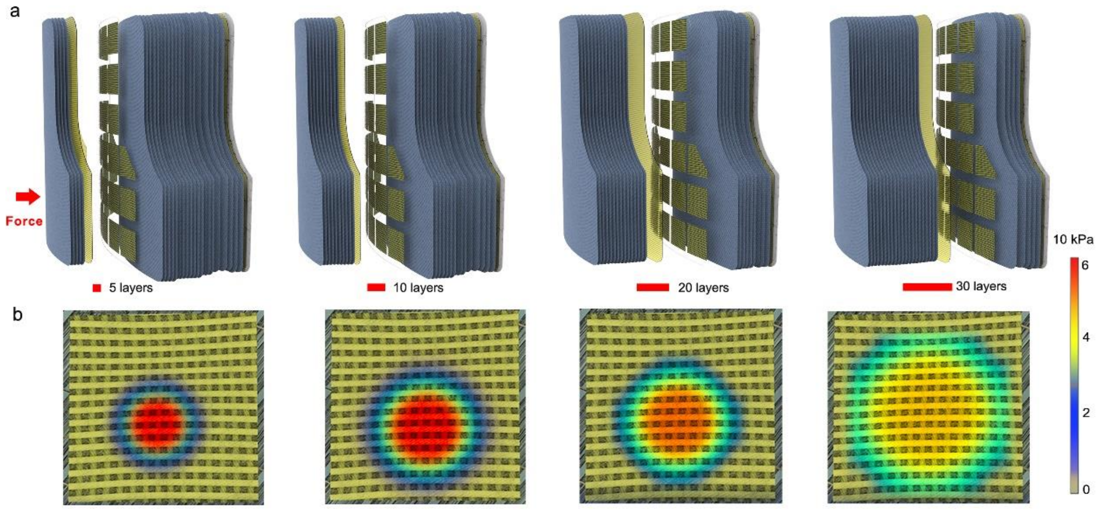


**Figure S11. Simulation of the relationship between the number of layers at the front-end of a single SP-ISFP and the stress.** a) Position model of 36 SP-ISFPs arrays in bulletproof vest. b) The stress distribution under different front-end layer numbers is simulated by finite element method.

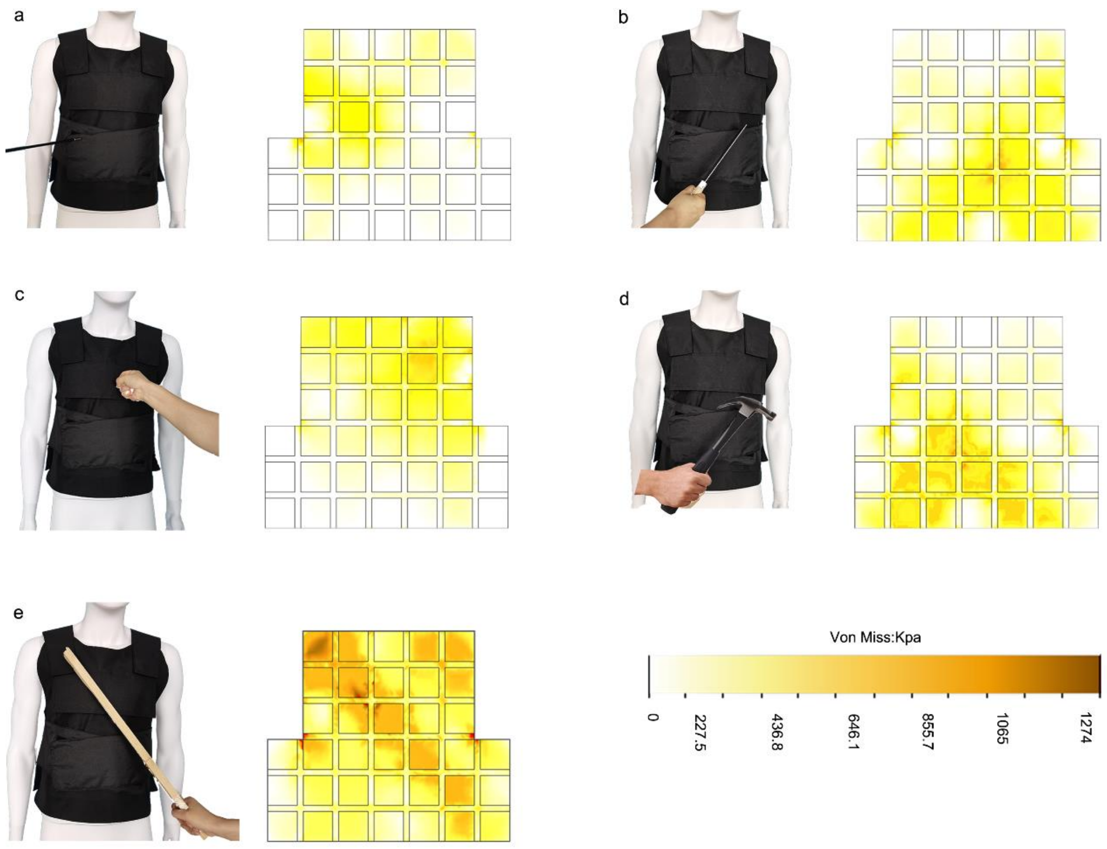


**Figure S12. Pressure distribution of the array of 36 SP-ISFPs, predicted by finite element analysis via SolidWorks software.** a) Being shot by an arrow. b) Being attacked by a knife. c) Be punched. d) Being struck by a hammer. e) Being hit by a stick.

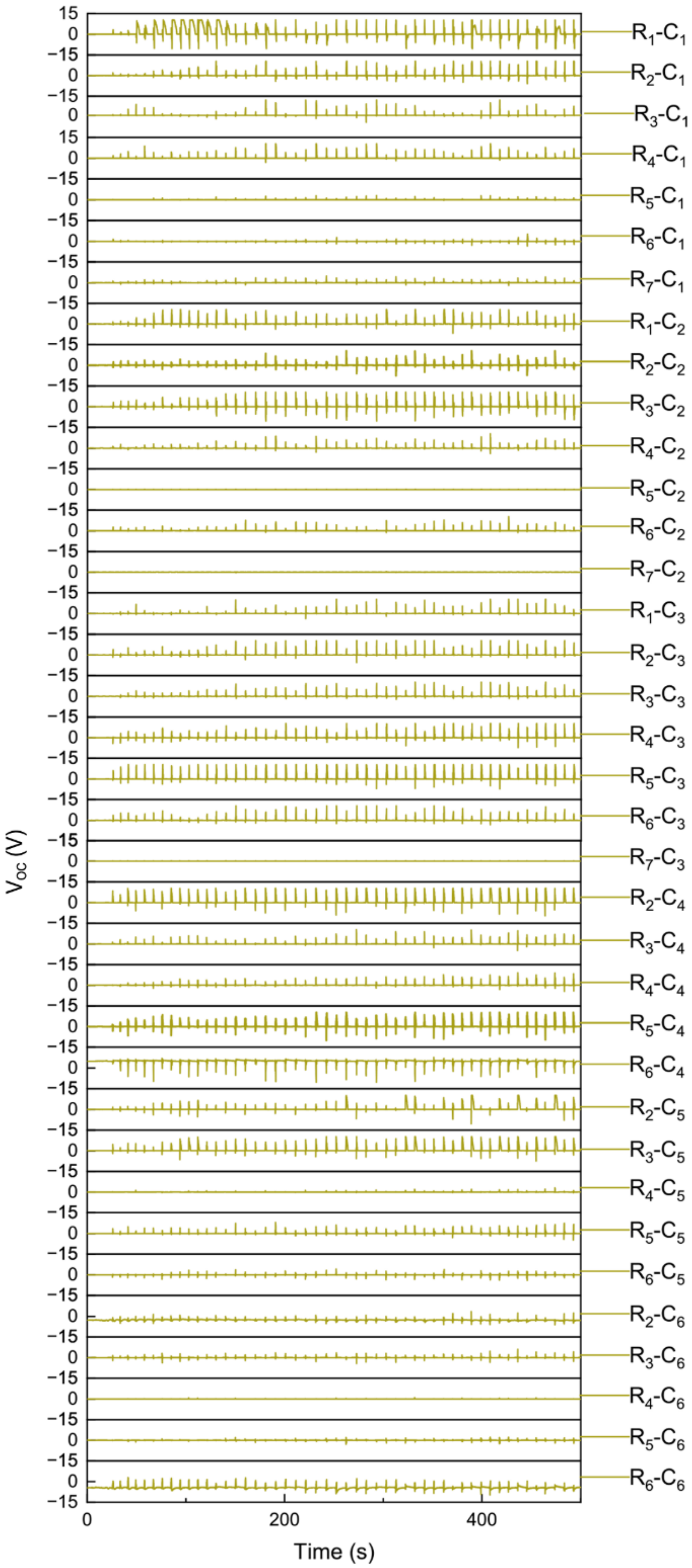


**Figure S13. The original waveform diagram of the stick acting fifty times.**

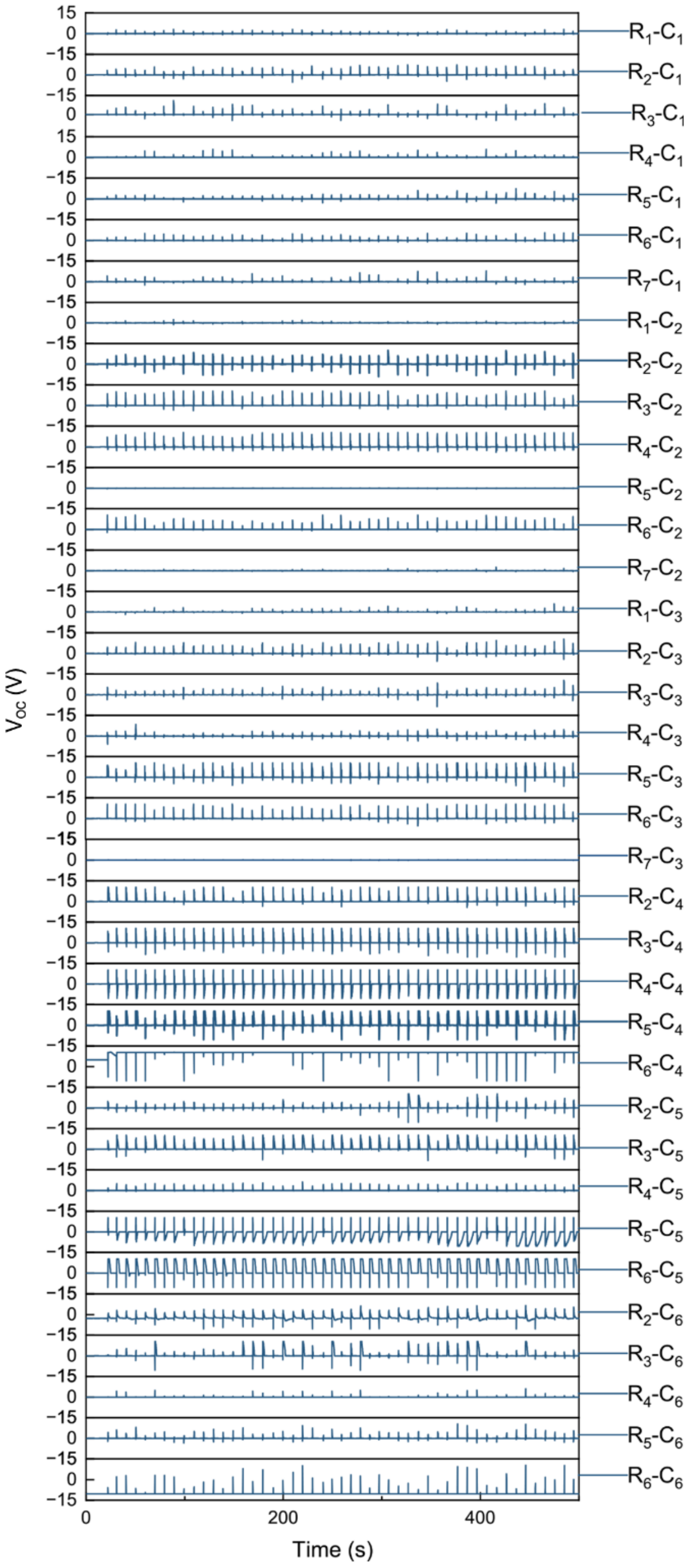


**Figure S14. The original waveform diagram of the hammer acting fifty times.**

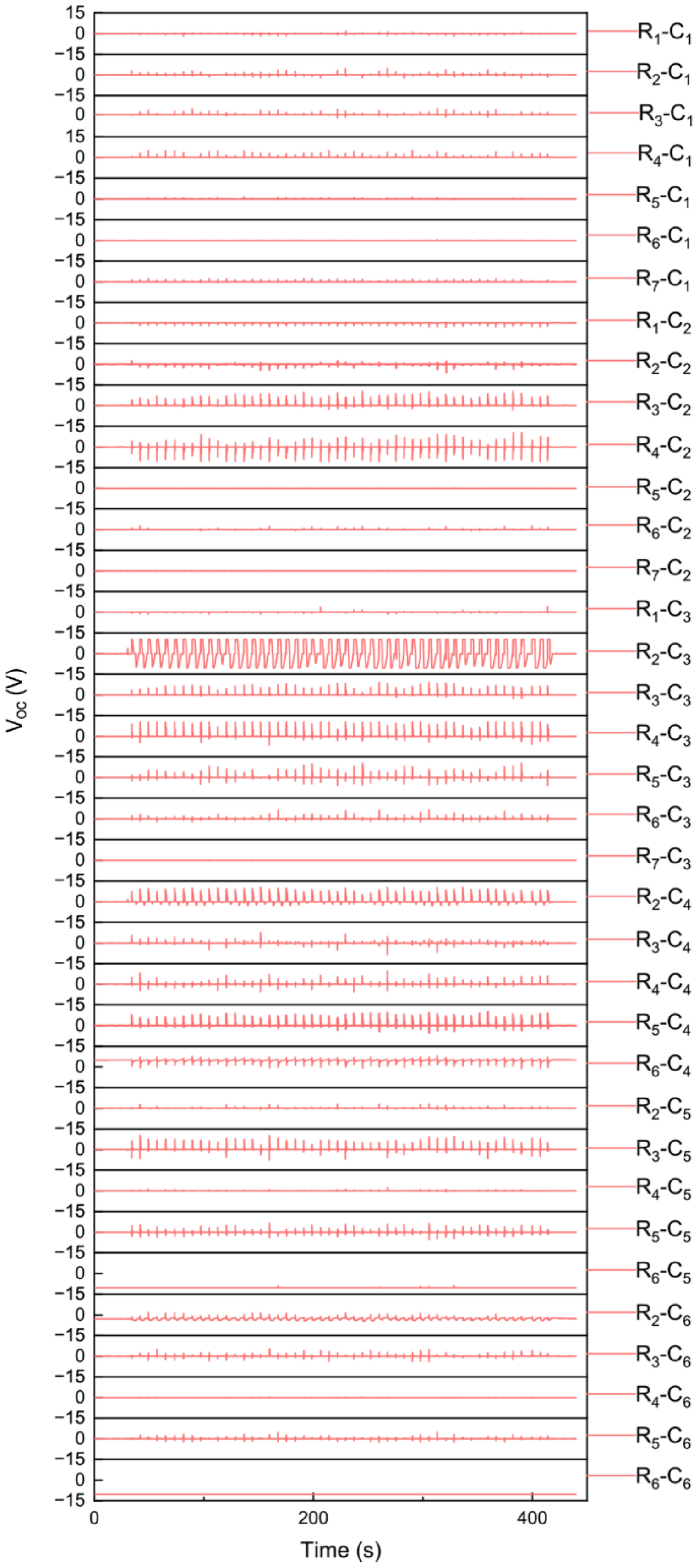


**Figure S15. The original waveform diagram of the arrow acting fifty times.**

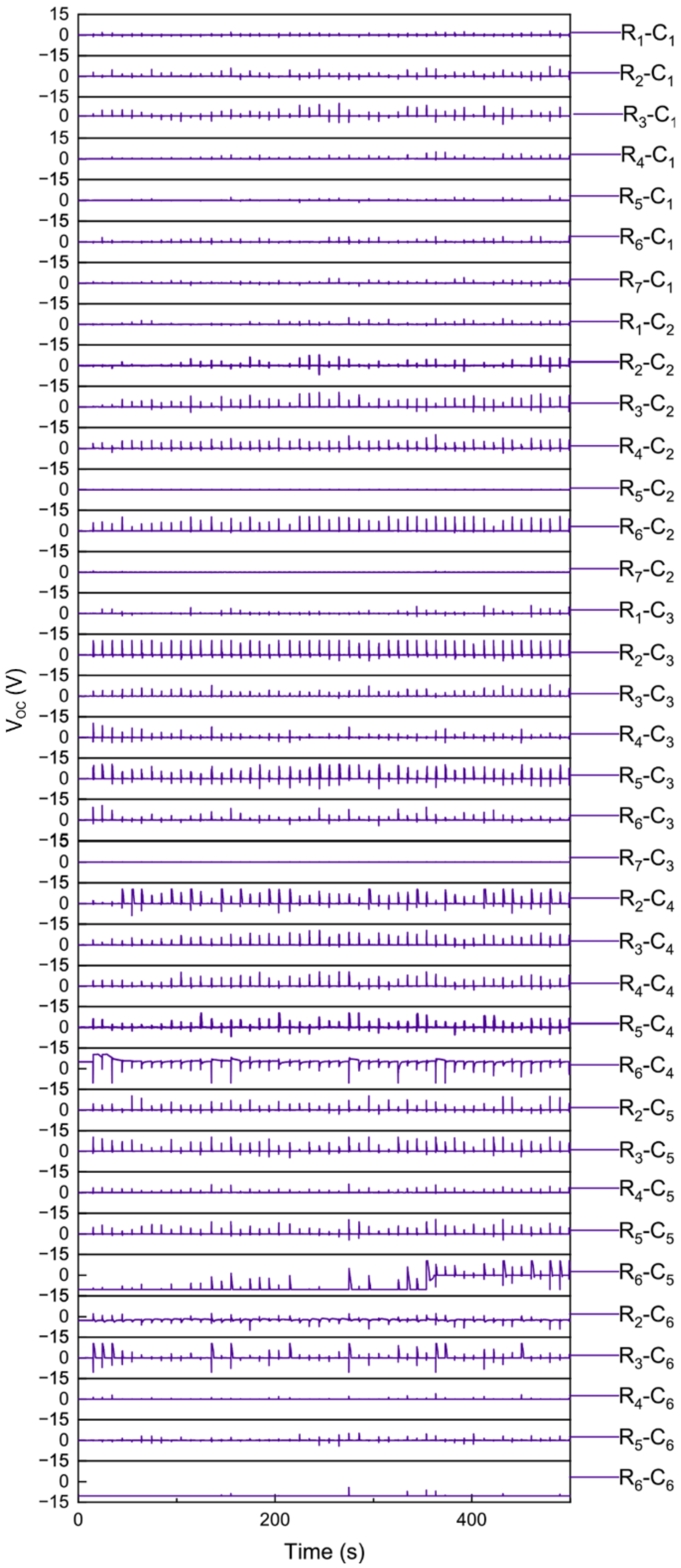


**Figure S16. The original waveform diagram of the fist acting fifty times.**

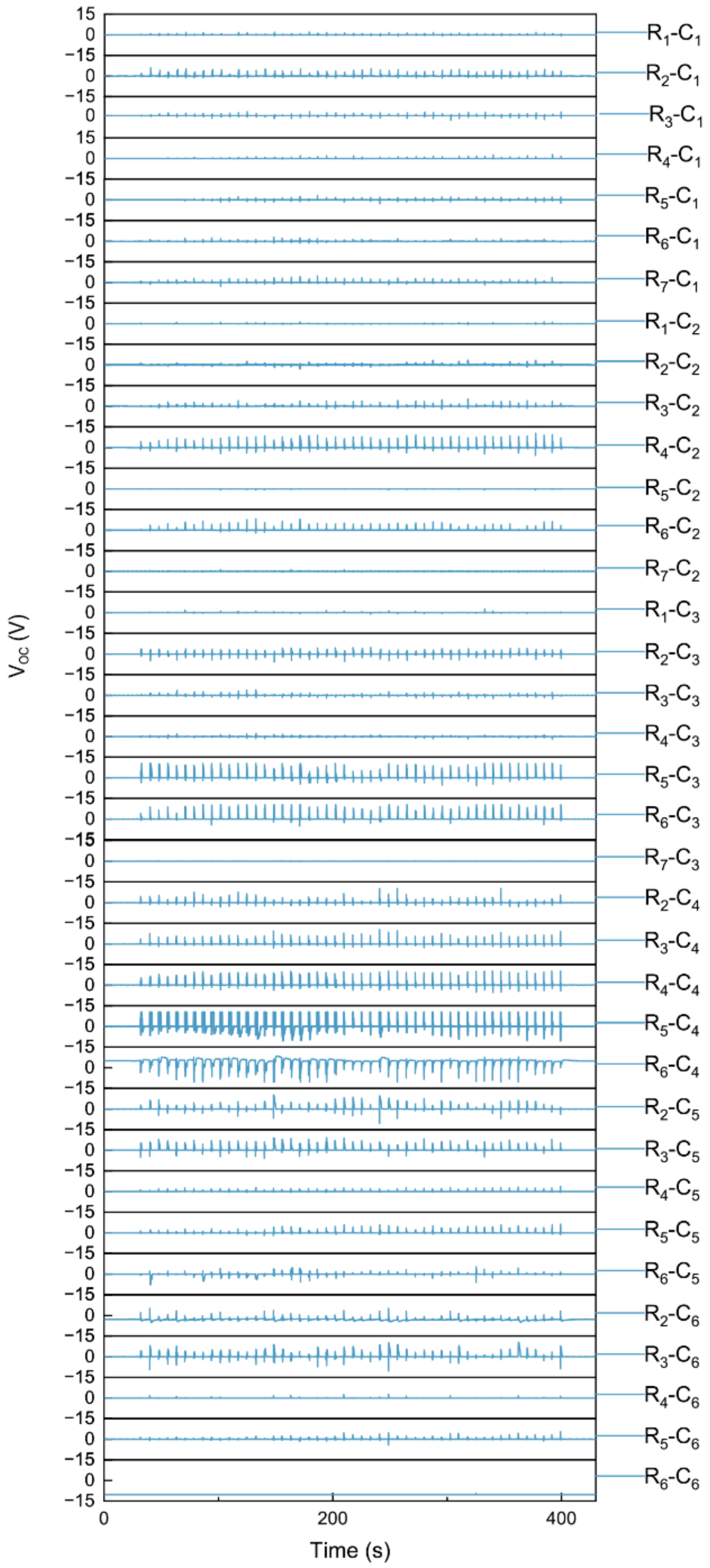


**Figure S17. The original waveform diagram of the knife acting fifty times.**

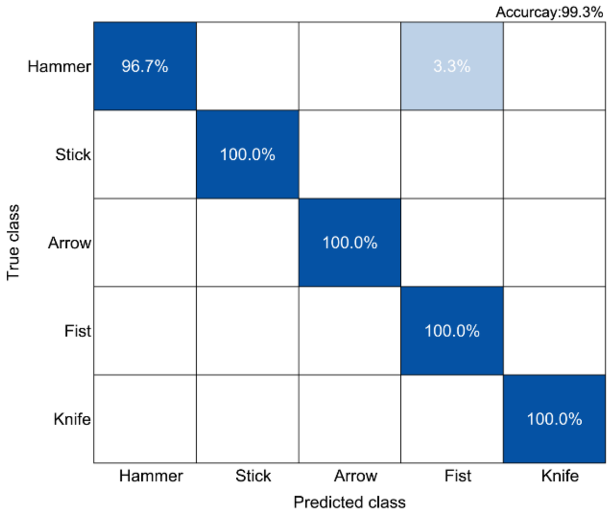


**Figure S18. Training set confusion matrix for weapon identification (accuracy of 98.7%).**

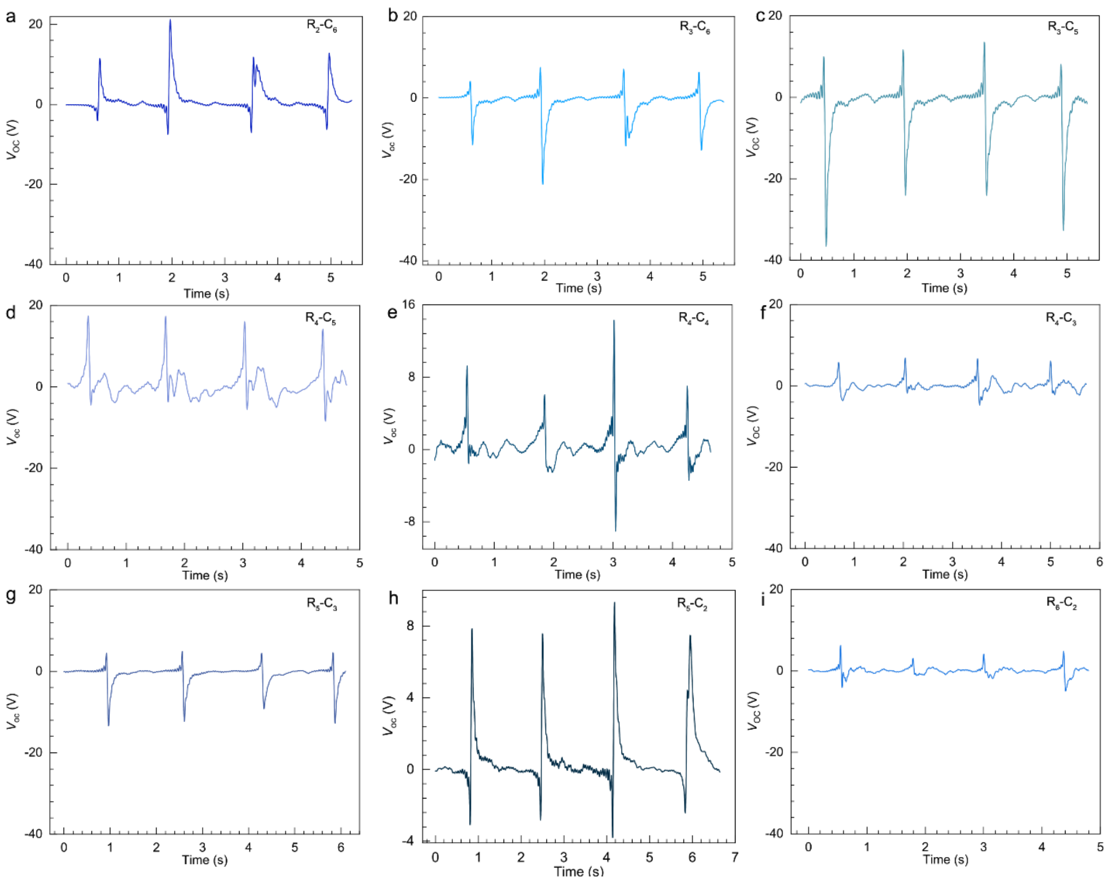


**Figure S19. Detail of SP-ISTPs voltage signal at 9 main force positions.** a) Row2-Column6. b) Row3-Column5. c) Row3-Column6. d) Row4-Column5. e) Row4-Column4. f) Row4-Column3. g) Row5-Column3. h) Row5-Column2. i) Row6-Column2

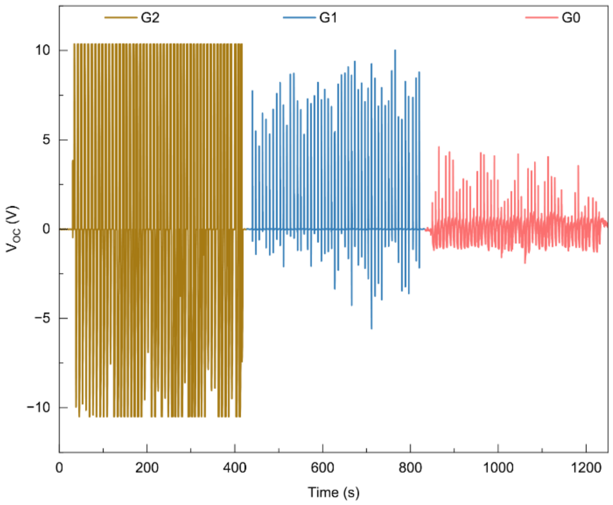


**Figure S20. Impact grade prediction waveform diagram.**

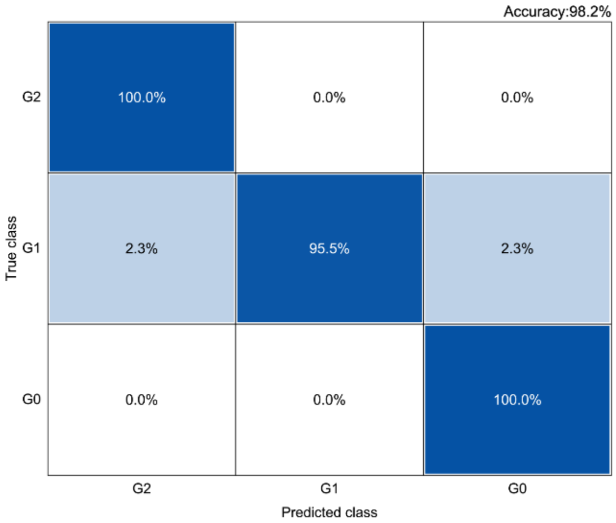


**Figure S21. Training set confusion matrix for impact grade judgment (accuracy of 98.2%).**
